# Brexit and the Constitutional Absence of England: Revisiting the English Question

**DOI:** 10.1093/ojls/gqag011

**Published:** 2026-02-26

**Authors:** Darryn Nyatanga

**Keywords:** English question, Brexit, devolution, territorial constitution, intergovernmental relations

## Abstract

This article re-examines the English Question in light of Brexit and the English Devolution and Community Empowerment Bill (DCEB). It argues that Brexit transformed England’s long-standing constitutional absence from a manageable anomaly into a source of acute constitutional strain. With the external disciplines associated with EU membership removed, the UK’s internal asymmetries have been exposed with greater force, revealing how England’s lack of distinct institutional representation weakens domestic accountability and complicates the operation of territorial governance. Through an analysis of the DCEB—the most ambitious attempt yet to rationalise England’s subnational governance—the article assesses whether recent reforms move England from administrative delegation towards constitutional accommodation. Applying four constitutional tests—identity, entrenchment, fiscal autonomy and voice—it concludes that while the DCEB enhances coherence and makes England’s subnational architecture more visible within the Union, it stops short of devolution in any meaningful constitutional sense. England’s governance remains derivative of Westminster authority, its institutions empowered yet fiscally dependent, politically included yet constitutionally insecure. The result is a more orderly form of constitutional absence: a settlement that manages England’s diversity without providing a collective locus of representation, which leaves unresolved the deeper question of how the largest nation in the Union can be governed without being constitutionally acknowledged. More fundamentally, it leaves unresolved how the Union can remain stable when its largest constituent nation is governed through undifferentiated state institutions rather than recognised channels of self-government.

## 1. Introduction

Writing in 2000, John Mason argued that ‘the present arrangements in England are inherently unstable and will lead to pressures for further change’.^1^ His warning went largely unheeded at the time, as England’s position within the UK’s constitutional framework remained characterised by what some have described as ‘the elephant in the room that we constantly ignore’.^2^ Since the establishment of devolution in 1998, this constitutional silence has persisted, rendering England the most conspicuous omission in the devolution settlement. In contrast to Scotland, Wales and Northern Ireland, each equipped with devolved legislatures and executives, England lacks its own political institutions. Instead, the UK Parliament and the UK government function as England’s domestic legislature and executive, respectively.

This article contends that this lacuna is not merely a procedural irregularity, but a structural feature with destabilising consequences. England’s lack of constitutional recognition creates two interrelated problems: one for England itself and one for the Union as a whole. For England, it produces a democratic deficit and blurs accountability in domestic governance. For the Union, it entrenches asymmetry, distorts intergovernmental relations and renders the exercise of England’s political dominance opaque rather than accountable. The English Question is therefore not simply a regional concern, but a constitutional imperative at the heart of the post-Brexit state.

There has, in recent years, been a notable shift in how this issue is framed within constitutional discussion. The House of Lords Select Committee on the Constitution, in its 2016 report on the Union and devolution, acknowledged that ‘the governance of England is becoming a key concern for those considering the territorial constitution’.^3^ Likewise, the House of Commons Public Administration and Constitutional Affairs Committee (PACAC), in its October 2022 report on English devolution, identified England’s current arrangements as ‘piecemeal and uncoordinated’, concluding that there is ‘an urgent and pressing need for significant reform’.^4^ What had long been treated as a technical omission is now increasingly recognised as a constitutional vulnerability.

This renewed attention reflects a deeper constitutional tension long recognised but never resolved. Over 50 years ago, the Kilbrandon Commission concluded that a federation of four units—England, Scotland, Wales and Northern Ireland—would be ‘so unbalanced as to be unworkable’,^5^ given England’s overwhelming size and wealth. Yet, Neil MacCormick’s later account of the ‘incorporating union’ suggested that asymmetry need not be fatal if embedded within a framework of mutual recognition and constitutional consent.^6^ This article situates the English Question within that unresolved tension: whether England’s scale renders its accommodation inherently destabilising, or whether continued non-recognition merely displaces rather than constrains its dominance.

The constitutional repositioning of England is closely tied to Brexit. The 2016 referendum marked a rare moment when English voters expressed a distinct constitutional will outside the context of a general election. With 53.4% of voters in England supporting Leave, England became the decisive voice in a UK-wide result that sharply diverged across its constituent nations.^7^ Yet, unlike Scotland, Wales or Northern Ireland—where equivalent constitutional moments yielded new institutions—England’s own ‘constitutional moment’ passed without recognition. Brexit thus intensified, rather than resolved, the long-standing ambiguity surrounding England’s constitutional status. The consequences of this absence have since deepened. The UK Internal Market Act 2020 (UKIMA) and post-Brexit intergovernmental reforms have introduced centralising pressures that disproportionately privilege English regulatory preferences while diminishing devolved autonomy.

Against this backdrop, the English Devolution and Community Empowerment Bill (DCEB) marks the most ambitious attempt in decades to restructure how England is governed internally. Promising to replace the fragmented model of ‘devolution by deal’, the Bill sets out a vision for extending statutory devolution across England by establishing combined county authorities (CCAs). These are to be led, where possible, by directly elected mayors, with devolved responsibilities in transport, housing, skills and economic development. The Bill marks a significant political shift, acknowledging that England’s governance cannot be sustainably managed through *ad hoc* negotiations or Westminster’s dual role alone. It reflects an emerging recognition that England’s internal governance requires greater coherence, visibility and co-ordination. However, as this article will argue, while the Bill introduces welcome institutional coherence, it falls short of resolving the deeper constitutional dilemma at the heart of the Union: the absence of a clear, equitable and enduring settlement for England within the UK’s asymmetrical and post-Brexit territorial constitution.

This article argues that the implications of Brexit and the constitutional reforms it has provoked require the UK to confront and resolve the English Question. That question comprises two principal dimensions: how England is governed internally and what role it plays within the UK’s evolving territorial constitution. While both are important, this article focuses on the first: the absence of a coherent and democratically accountable system of English governance. It argues that the failure to accommodate England institutionally is not merely a symbolic omission, but a constitutional deficiency with material consequences for the legitimacy and durability of the post-Brexit constitution. Furthermore, the article argues that resolving the English Question is not merely necessary to placate dissatisfaction in the devolved jurisdictions, but is vital for England itself. England’s lack of institutional representation renders it structurally disadvantaged in three ways: it dilutes democratic accountability over English domestic policy; it conflates English interests with UK-wide ones, distorting national policy making; and it leaves England without a voice in intergovernmental processes where other parts of the UK are directly represented. Reform, therefore, is not a concession to others, but a constitutional necessity for England’s own coherence, accountability and capacity for self-government.

### A. Literature Review

A substantial body of scholarship has explored the English Question, typically framed through the lens of constitutional asymmetry and the West Lothian Question. Vernon Bogdanor and Robert Hazell laid the groundwork for recognising the democratic anomalies inherent in England’s non-devolved status.^8^ More recently, scholars such as Michael Kenny, Iain McLean and Akash Paun have examined England’s governance from regional and decentralist perspectives, advocating for stronger subnational institutions to mitigate the centralisation of UK politics.^9^

Stephen Tierney has advanced the understanding of UK devolution as quasi-federal in logic if not in form, emphasising shared rule and self-rule dynamics that mirror those of federations. However, Tierney stops short of prescribing institutional reforms specific to England, instead urging recognition of federal principles within the existing territorial constitution.^10^

This article builds on and departs from that literature by situating the English Question squarely in the post-Brexit constitutional context. It highlights how Brexit, UKIMA and the evolving intergovernmental landscape have intensified the costs of England’s constitutional invisibility. In doing so, it combines doctrinal legal analysis with practical policy developments, making the case for a systemic accommodation of England within the UK’s territorial constitution.

### B. Scope and Methodology

This article adopts a legal-institutional approach to the English Question. Rather than engaging with the cultural or sociological dimensions of English national identity, it focuses on the structural and constitutional features of governance. Through analysis of legislation, intergovernmental arrangements, parliamentary practice and legal scholarship, the article traces how England’s institutional invisibility has generated democratic and constitutional consequences in the wake of Brexit.

The article proceeds as follows. It first traces the conceptual evolution of the English Question within the UK’s asymmetrical devolution framework, evaluating earlier constitutional responses such as regional assemblies and English Votes for English Laws. It then assesses how Brexit has reframed the issue, with particular attention to the UK Internal Market Act 2020 and shifting dynamics of intergovernmental relations. Next, it examines the current reform landscape, focusing on the English Devolution and Community Empowerment Bill and Labour’s proposals for regional devolution. The conclusion reflects on the constitutional imperative of resolving the English Question and the risks of continued institutional absence.

## 2. The English Question: Origins and Evolution

### A. The West Lothian Question and Constitutional Asymmetry

In contrast to the devolved territories of Scotland, Wales and Northern Ireland, England lacks constitutional recognition within the UK’s territorial constitution and possesses no coherent system of devolution at either the regional or local level. This constitutional patchwork has meant that England continues to be governed directly by the central institutions of the UK—principally Parliament and the UK government. As a result, England’s lack of distinct institutional representation has allowed the UK Parliament to continue functioning as a unitary legislature, thereby sustaining the doctrine of parliamentary sovereignty in its most centralised form. Since England comprises the majority of the UK’s population and territory, the UK Parliament’s authority over English domestic matters appears naturalised, reaffirming England’s dominance within the Union. At the same time, this arrangement conceals the fact that England is the only constituent part of the UK without its own devolved institutions, reinforcing England’s ‘constitutional invisibility’.

This constitutional arrangement has repeatedly prompted fundamental questions: where does England fit within the UK’s constitutional framework, and how should England be governed in an era when significant powers have been devolved to Scotland, Wales and Northern Ireland? These questions and the problems they reveal are collectively understood as the English Question. As identified by the House of Lords Constitution Committee in its 2016 report, *The Union and Devolution*, the English Question encompasses two principal constitutional facets: ‘the representation of England as a whole, and devolution or decentralisation to regional or local levels within England’.^11^ The first relates to England’s formal recognition within the UK’s territorial constitution; the second to the governance of England itself. Together, these dimensions trace the contours of a problem that has become increasingly difficult to ignore, particularly in the wake of Brexit.

The historical genesis of the English Question is often linked to the West Lothian Question, famously articulated by Tam Dalyell, then Labour MP for West Lothian, during a House of Commons debate in 1977. Anticipating the effects of devolution, Dalyell asked:

For how long will English constituencies and English honourable Members tolerate not just 71 Scots, 36 Welsh and a number of Ulstermen but at least 119 honourable Members from Scotland, Wales and Northern Ireland exercising an important, and probably often decisive, effect on English politics while they themselves have no say in the same matters in Scotland, Wales and Ireland?^12^

This perceptive question captured a constitutional asymmetry that would only become more pronounced after the enactment of the Scotland Act 1998, the Government of Wales Act 1998 and the Northern Ireland Act 1998. Ironically, Dalyell’s concern predated the implementation of devolution, but it anticipated the legislative and procedural issues that followed. The problem he identified—MPs from devolved jurisdictions voting on England-only matters—remains unresolved.

In the late 1970s, Lord Irvine, the inaugural Lord Chancellor under the Blair government and a key architect of its constitutional reform agenda, is reported to have remarked that ‘the best thing to do about the West Lothian question is to stop asking it’.^13^ For a time, that strategy appeared politically viable. The defeat of the 1979 devolution referendums in Scotland and Wales, where neither proposal met the required thresholds, allowed the incoming Conservative government under Margaret Thatcher to declare the matter settled ‘for a generation’.^14^ Thus, the question of England’s role within the UK’s emerging territorial constitution was politically avoidable, if not constitutionally resolved.

The election of New Labour in 1997 fundamentally altered this trajectory. As part of its sweeping programme of constitutional reform, the Labour government introduced devolution to Scotland and Wales through the Scotland Act 1998 and the Government of Wales Act 1998, with power-sharing reintroduced in Northern Ireland under the Northern Ireland Act 1998. However, no equivalent constitutional settlement was offered to England. Instead, England was left under direct Westminster rule, with only a piecemeal experiment in regional decentralisation via the Greater London Authority Act 1999, which established a mayor and assembly for London. These developments revived the West Lothian Question in tangible terms. What had once been a theoretical parliamentary puzzle now became a practical constitutional anomaly, sharpening debates over England’s governance and status within the UK.

The asymmetrical evolution of devolution has been a decisive factor in the establishment and continual re-emergence of the English Question. The House of Commons’ PACAC report on English devolution observed that ‘the more the institutions in devolved nations have become an established part of the UK’s constitutional architecture, the more awkward and potentially problematic the position of England becomes’.^15^ Scottish devolution, in particular, has repeatedly intensified debates around England’s position. Following the 2014 Scottish independence referendum, which addressed Scotland’s long-standing constitutional aspirations, then Prime Minister David Cameron remarked: ‘I have long believed that a crucial part missing from this national discussion is England … We have heard the voice of Scotland—and now the millions of voices of England must also be heard.’^16^

This statement brought the English Question back onto the political agenda, highlighting England as the overlooked component of the UK’s territorial constitution.

### B. Pre-Brexit Constitutional Responses: Regional Assemblies, Devolution Deals and English Votes for English Laws

In the years preceding Brexit, successive UK governments grappled with the constitutional awkwardness of England’s position through a range of institutional experiments, each reflecting different conceptions of how best to reconcile England’s governance with the broader asymmetrical structure of the Union. These initiatives—namely, Labour’s proposals for regional assemblies, the devolution deals system advanced by the Coalition and Conservative governments, and the procedural innovation of English Votes for English Laws (EVEL)—represented distinct attempts to navigate the constitutional pressures generated by devolution elsewhere. Yet, each initiative failed, for different reasons, to provide England with either a stable constitutional identity or a sustainable structure of representation. Taken together, these experiments reveal a pattern of constitutional improvisation without constitutional design, the legacy of which shapes the DCEB now before Parliament.

### C. Regional Assemblies: Devolution without a Demos

New Labour’s attempt to establish elected regional assemblies stands as the most ambitious effort to tackle one facet of the English Question—namely, the governance of England. This proposal aimed to avoid the risks of creating an English Parliament that might rival Westminster and the UK government, due to England’s demographic scale.^17^ Instead, it sought to disperse power within England itself by establishing assemblies across eight English regions, modelled in part on devolved structures established in Wales in 1998.

Labour’s approach was first noted in its 1997 manifesto promise to allow ‘people, region by region, to decide in a referendum whether they want directly elected regional government’.^18^ This culminated in the 2002 White Paper, *Your Region, Your Choice*, which outlined plans for regional assemblies with responsibilities over transport, housing, strategic planning, and aspects of economic development.^19^ The Draft Regional Assemblies Bill 2004 set out a framework under which each assembly would have between 25 and 35 elected members (via the Additional Member System used in Wales) and modest executive powers. Importantly, implementation required popular endorsement through referendums.

Alongside this political agenda, the government had already established regional development agencies (RDAs) under the Regional Development Agencies Act 1998. These bodies operated in each of the eight English regions outside London and were tasked with promoting economic development, competitiveness, employment and sustainable growth. Although unelected, RDAs represented a significant institutional innovation, serving as regional interlocutors between central government and local economies. Their mandates included the co-ordination of EU structural funds, regional economic planning, and oversight of skills and training strategies. RDAs were intended to act in concert with future elected regional assemblies, forming a two-tier regional governance model that combined democratic accountability with technocratic delivery. In this sense, RDAs were not merely economic actors, but institutional stepping stones towards a more integrated model of English regionalism.^20^

From inception, however, the model combined administrative cartography with thin constitutional theory. Three structural weaknesses were baked in. First, identity. The assemblies were intended to be representative forums for regional self-government, yet the regions themselves had shallow democratic roots. The boundaries owed more to Whitehall rationality and EU Nomenclature of Territorial Units for Statistics geographies than to settled political communities capable of underwriting a durable mandate. In the absence of a thick narrative of regional *demos*—of shared identity, stakes and future—the policy struggled to persuade voters that a new representative tier would add voice rather than bureaucracy. This difficulty contrasts with, but should not be overstated in light of, the experience of Scottish and Welsh devolution. In neither case did a fully articulated political identity simply precede institutional creation. Welsh devolution, in particular, was endorsed by a narrow referendum majority and only gradually acquired popular attachment as institutions took root. The more salient distinction lies in scale and consequence. In Scotland and Wales, institutional development occurred within territorially bounded communities whose recognition did not reconfigure the balance of power within the Union. In England’s regions, by contrast, polity was expected to precede identity in a context where regional institutions lacked both historical depth and the capacity to command political loyalty independently of the central state.

Second, finance. The assemblies were designed with limited fiscal autonomy. They would rely on central transfers, with negligible or no own-source revenues. Without predictable revenue streams—let alone tax-varying powers—regional autonomy would be discretion-dependent and programme-driven. The optics were unforgiving: the scheme could be framed as an extra layer of politicians ‘funded from London’, not a locus of accountable, self-directed government. Parliamentary scrutiny of the Draft Regional Assemblies Bill confirmed the tightness of the purse and the deliberate limits placed on functional scope.^21^

Third, entrenchment. Constitutionally, assemblies were to be ordinary statutory bodies, established and shaped by primary legislation, but without any protection against later alteration or repeal by Westminster. In constitutional terms, this was statutory delegation rather than constitutional devolution. The absence of legal or conventional entrenchment weakened claims to autonomy: institutions that can be remodelled from the centre are hard to present as partners within a territorial constitution. The House of Lords Constitution Committee would later diagnose the broader syndrome: England’s governance had become ‘piecemeal and uncoordinated’, with little sense of stable constitutional allocation.^22^

The political reckoning came with the North East referendum on 4 November 2004. On a turnout of just 48%, voters rejected the assembly by 78% to 22%. This defeat was so decisive that the government abandoned planned referendums in the North West and in Yorkshire and the Humber.^23^ The collapse of the regional assemblies initiative decisively closed the door on one possible constitutional pathway for embedding England into the UK’s evolving territorial order.

Read against Kilbrandon and MacCormick, the regionalism episode is revealing. Kilbrandon’s classic concern was scale: a four-unit federation with England as one part would be ‘so unbalanced as to be unworkable’. Regionalism sought to evade that problem by shrinking England, transforming a single dominant unit into a set of smaller ones. Yet, MacCormick’s insight that stability in a multinational polity depends not on schematic symmetry but on consent between self-governing communities exposes the limits of this strategy. Redrawing territorial boundaries cannot, by itself, generate the political authority required for durable self-government.

This does not deny that institutions can sometimes precede, and even cultivate, political voice. Welsh devolution demonstrates that polity and identity need not emerge in a settled sequence, and that popular attachment may deepen only gradually after institutions are created. Northern Ireland, by contrast, illustrates that constitutional form can exist in the absence of shared political consent, producing institutions that manage division rather than resolve it. The regional English experience points to a different, but related, difficulty. Where identity is weak, finance is dependent and status is constitutionally insecure, institutional creation by administrative means is unlikely to generate either durable legitimacy or popular ownership. In this sense, regionalism did not fail because constitutional voice must always precede constitutional form, but because form was offered without the political, fiscal and constitutional conditions through which voice might plausibly develop.

For present purposes, the DCEB inherits this history in three ways. (i) Identity: proposed CCAs and any strategic authorities (SAs) echo the earlier reliance on constructed units; unless linked to recognisable communities of interest and given agendas that matter (eg transport integration, skills, housing affordability), they risk the same legitimacy deficit. (ii) Finance: if revenue remains predominantly Treasury-controlled and time-limited, the assemblies-by-another-name will struggle to demonstrate meaningful autonomy—precisely the referendum-era critique. (iii) Entrenchment: without heightened legal protection (eg codified constraints on ministerial alteration), today’s framework may perfect the machinery of regional administration while still failing to constitute regional self-government. The regionalism episode thus supplies the DCEB’s test: can the Bill convert administrative regions into constitutional polities by grounding them in identity, finance and status, rather than in cartography alone?^24^

### D. Devolution Deals and Metro Mayors: Localism without Constitutionalism

Following the collapse of the regional assemblies initiative, the constitutional focus shifted towards more flexible and pragmatic models of decentralisation. The incoming Coalition government in 2010 deliberately moved away from Labour’s regional scale, instead promoting a ‘localism’ agenda designed to empower functional economic areas such as cities and counties. The Coalition government’s constitutional instincts were centralising in philosophy but decentralising in rhetoric. The new settlement was designed not to redistribute sovereignty, but to delegate responsibility within a tightly managed framework of intergovernmental control.

The legal framework for this model was first established in the Local Democracy, Economic Development and Construction Act 2009, which enabled groups of local authorities to form combined authorities with joint responsibility for areas such as transport and economic development. The Localism Act 2011 made modest but significant amendments to this framework, supporting the broader agenda of empowering local institutions and introducing a general power of competence for local authorities. However, it was the Cities and Local Government Devolution Act 2016 that significantly expanded the scope of the 2009 Act. This legislation allowed for directly elected mayors, the conferral of additional powers on combined authorities through secondary legislation and a more structured mechanism for devolving a wider range of policy competences—including policing, housing, and aspects of health and skills policy.

Under this framework, local authorities were invited to form combined authorities, which could then receive specific competences through negotiated devolution deals. Many of these new bodies were led by directly elected metro mayors. The powers devolved varied significantly by region and deal, but typically included responsibilities over transport, housing, planning, adult skills provision and economic development. In some cases, more extensive powers were conferred, such as oversight of local policing or elements of healthcare policy, though these remained exceptional. At its strongest, this model was exemplified by Greater Manchester, whose combined authority evolved through successive agreements to acquire powers over police and crime, health and social care integration, and fiscal policy levers.

Overall, the political logic of these ‘devolution deals’ was pragmatic and asymmetrical. Rather than an open constitutional design, reform proceeded by bilateral negotiation between local coalitions and Whitehall. Powers were transferred by statutory instrument, not by constitutional right, and often with complex caveats about policy domains and financial liability. In form, this was a continuation of English constitutional exceptionalism: incrementalism and discretion in place of codified authority. In substance, it amounted to local administration by contract, rather than self-government by constitutional principle.^25^

The governance consequences were mixed. On the one hand, the model generated new forms of territorial leadership and visible accountability in English city regions. The direct election of mayors in Greater Manchester, Liverpool, the West Midlands and elsewhere created political figures capable of articulating local interests within national debates. In doing so, these institutions achieved what the abortive regional assemblies could not—recognition. As Michael Kenny and Tom Kelsey have argued, the metro mayors became a new class of political actor who could personify and champion local interests in the national political arena.^26^ They gave England a vocabulary of regional representation that had previously been absent. On the other hand, these very successes exposed the system’s constitutional fragility. Beneath the optics of empowerment lay four persistent deficits: of voice, finance, status and system.

First, the voice deficit. Prior to the recent creation of the Council of the Nations and Regions, combined authorities articulated local priorities but had no standing in the UK’s intergovernmental system.^27^ Unlike Scotland, Wales and Northern Ireland, whose executives have always participated formally in intergovernmental relations, English mayors were structurally excluded. As the House of Lords Constitution Committee observed, ‘England as a whole has no institutional expression in intergovernmental relations; it is represented only through the UK Government’.^28^ As observed during the COVID-19 pandemic, metro mayors could speak loudly, but they could only do so from outside the constitutional tent.

Second, the finance deficit. The fiscal dimension of English devolution remains entirely dependent on the Treasury. Combined authorities receive funding through short-term ‘growth deals’, competitive pots and capped precepts. PACAC, in its 2022 English devolution report, described these arrangements as ‘piecemeal and uncoordinated’, creating a system in which local budgets are contingent on Whitehall discretion rather than locally raised revenues.^29^ The contrast with the devolved nations is stark: Scotland and Wales possess block grants and limited tax-raising powers; English regions operate on borrowed autonomy.

Third, the status deficit. Constitutionally, the model remains delegatory. Powers can be expanded, reduced or revoked by ministerial order under section 105 of the Local Democracy, Economic Development and Construction Act 2009, as amended by the 2016 Act. There is no statutory guarantee of permanence or entrenchment. Mark Elliott has observed that, absent either referendum endorsement or constitutional-statute recognition, English devolution rests on a revocable grant, not a reserved right.^30^ The distinction between delegation and devolution is not semantic—it determines whether autonomy is politically durable or legally contingent.

Fourth, the system deficit. Coverage is uneven. Some areas of England enjoy complex multi-tier arrangements; others remain entirely outside the system. The outcome is what can be described as a postcode constitution, where the scope of local power is determined by geography and negotiation rather than principle.^31^ The map of English self-government thus reproduces inequality under the guise of experimentation: Manchester governs; Cornwall negotiates; Cumbria waits.

The logic of the devolution deals was therefore administrative differentiation, not constitutional transformation. They created room for innovation but not recognition; flexibility but not security. Stephen Tierney’s distinction between *shared rule* and *self-rule* in quasi-federal systems is instructive here: England’s regions were given elements of self-rule but denied any institutionalised role in shared rule.^32^ The absence of a federal or even formalised intergovernmental chamber meant that local voices cannot influence UK-wide decisions that directly shape their competences—an asymmetry that Brexit later made explicit.

Read in comparative context, the English ‘localism’ model exemplifies what scholars of multilevel governance describe as decentralisation without constitutionalism. Powers exist, but principles do not. The result, as Vernon Bogdanor has observed, is that ‘England remains the only country in Europe without its own system of devolved government’.^33^

The implications for the DCEB are immediate. The Bill builds directly upon the devolution-deal legacy, seeking to regularise the architecture by converting combined authorities into CCAs and embedding the mayoral model across England. Yet, unless it addresses the four deficits identified above, it risks reproducing them in statutory form: visible institutions without constitutional guarantees.

For the DCEB to mark a constitutional step rather than another administrative cycle, three reforms appear essential. First, entrenchment—CCAs should enjoy protection akin to the devolved settlements, at least through recognition as constitutional statutes immune from implied repeal. Second, fiscal autonomy—a stable share of revenue, or new taxing powers, is necessary to translate competence into agency. Third, integration—English regional leaders require a structured role within intergovernmental relations. This reform proposal is already taking shape through the recent creation of the Council of the Nations and Regions, which held its first meeting in late 2024. This forum brings together the prime minister, devolved leaders and English metro mayors. It is too early to tell if this new forum will address the issue of integration fully. Overall, only with these safeguards could the DCEB transform the logic of English decentralisation from one of ministerial concession to one of constitutional partnership.

### E. English Votes for English Laws: Procedure as Proxy

If regional assemblies and devolution deals were exercises in administrative devolution, EVEL represented an attempt at procedural containment—a parliamentary device designed to address England’s constitutional anomaly without altering the territorial architecture of the Union. Where the earlier experiments sought to distribute power geographically, EVEL sought to differentiate competence procedurally, within the framework of Westminster itself.

The immediate antecedent was the McKay Commission (2013), established by the Coalition government to examine the consequences of devolution for the House of Commons.^34^ Its central recommendation was modest but symbolically potent: ‘decisions with a separate and distinct effect for England (or for England and Wales) should normally be taken only with the consent of a majority of MPs sitting for constituencies in England (or England and Wales)’. The Commission rejected an English Parliament as politically unworkable and instead proposed a rule of double consent—preserving the Commons as a unitary chamber while recognising England’s distinct legislative interest.

In July 2015, the Conservative government implemented the Commission’s proposal through a series of Standing Order amendments. The new procedures created a two-stage legislative process for ‘England-only’ or ‘England and Wales-only’ bills and clauses: first, certification by the Speaker under Standing Order No 83J; and second, an England-only legislative grand committee, composed of English MPs, empowered to consent or veto before final Third Reading.^35^ This was, in form, an internalisation of the territorial logic of devolution—England’s representation brought home to Westminster.

Yet, EVEL was constitutional bricolage: it treated the symptom procedurally while leaving the structure intact. The measure’s virtues lay in symbolism, not substance. It neither created an English forum nor endowed England with a representative executive. Its innovation was to make certain votes contingent on English majorities, but this adjustment operated within the same institutional shell that had long conflated England’s and the UK’s interests. Three limitations defined its short life.

First, opacity. The certification process was notoriously complex. Determining whether a Bill ‘related exclusively’ to England or to ‘England and Wales’ required intricate cross-referencing of schedules and statutes. The Speaker’s rulings became quasi-judicial exercises in constitutional geography, largely invisible to the public and understood by few MPs. This complexity undermined the very purpose of the reform—transparent accountability for England-only legislation.

Second, marginality. In practice, EVEL was rarely consequential. Between 2015 and its suspension in 2020, only a handful of Bills were certified as England-only, and in none did the English veto alter the outcome.^36^ The measure’s redundancy reflected both the political arithmetic of Conservative majorities and the structural reality that most legislation, while primarily English in effect, carried UK-wide financial or administrative implications. As the House of Commons Library later noted, ‘the boundaries of “England-only” competence proved more elusive than anticipated’.^37^

Third, fragility. Procedurally, EVEL rested entirely on standing orders, not statute. It could be suspended or repealed by a simple majority, and the government did precisely that in July 2021, citing the need for ‘greater flexibility’ and the ‘successful operation of the Union’.^38^ Its demise passed with barely a parliamentary murmur, underscoring that EVEL had generated no constitutional constituency of its own. In the end, it had provided England with a veto mechanism but not a voice, a safeguard without a forum.^39^

From a constitutional standpoint, EVEL preserved the formal integrity of the Commons but at the cost of perpetuating England’s constitutional invisibility. Where the devolution deals gave localities governance without self-government, EVEL gave England recognition without representation.

Read through the prism of Kilbrandon and MacCormick, EVEL demonstrates the UK’s continuing preference for *ad hoc* adaptation over deliberate settlement. Kilbrandon’s warning that a federation of four units would be ‘unbalanced and unworkable’ haunted the proposal implicitly: creating an English Parliament remained off-limits, so procedural improvisation filled the void. But MacCormick’s idea of the ‘incorporating union’ suggests that shared governance requires mutual recognition among distinct polities, not the internalisation of one within another. EVEL, by contrast, reasserted Westminster’s monistic logic under the veneer of territorial sensitivity.

The constitutional lessons for the DCEB are instructive. The Bill’s ambition to ‘empower’ English regions and localities is laudable, but unless accompanied by institutional mechanisms that give those entities a voice in shared rule, it may replicate EVEL’s deficiency on a broader scale: participation without parity. A coherent English settlement requires not merely decentralisation, but representation—structures through which English preferences can be articulated, co-ordinated and reconciled within the Union, rather than inferred through Westminster arithmetic.

The cumulative effect of England’s pre-Brexit experiments in territorial governance was to confirm a persistent constitutional reflex: incremental management of the English Question rather than its institutional resolution. Each reform responded to symptoms—representation, accountability, administrative efficiency—but none addressed the structural cause: the absence of a constitutional framework recognising England as a self-governing polity within a devolved Union.

Viewed together, these initiatives reveal a consistent constitutional pattern: delegation without devolution. Authority was devolved *to* regions or localities, but never *of* them; the UK government remained both principal and referee. Political caution rooted in Kilbrandon’s fear that an empowered England would destabilise the Union produced a cycle of half-measures. Yet, as Neil MacCormick’s theory of the ‘incorporating union’ suggests, stability rests not on suppressing asymmetry, but on recognising it within a framework of mutual consent. England’s omission from that framework is no longer sustainable.

The DCEB inherits this history in both promise and peril. It codifies decades of administrative improvisation, but whether it constitutes a constitutional settlement depends on its response to four interlocking tests derived from England’s earlier failures:

1. Can the Bill root CCAs and SAs in recognisable communities of interest, or will they remain technocratic constructs?
2. Will the new structures enjoy protection as constitutional statutes, immune from unilateral alteration, or remain subject to ministerial redesign?
3. Does the framework provide predictable, locally controlled revenue streams, or will ‘empowerment’ continue to depend on central grant funding?
4. Will England’s representation remain implicit in the UK government?

Absent progress on these fronts, the DCEB risks perfecting the machinery of administrative decentralisation while entrenching the substance of central control. Its success will turn not on the number of authorities created or mayors elected, but on whether England’s internal governance is finally endowed with constitutional standing—the very condition denied to its predecessors. The English Question cannot be deferred by design; it must be settled by recognition.

As the next sections will explore, Brexit has made the constitutional invisibility of England increasingly unsustainable, prompting a renewed debate over England’s governance. In this light, the failures and partial solutions of the past stand not merely as historical episodes, but as essential context for understanding the stakes and limitations of current and potential future reforms.

## 3. The Brexit Effect: Post-Brexit Constitutional Recalibration and the Imperative to Accommodate England

As the preceding analysis demonstrated, successive UK governments have largely approached the English Question from the vantage point of their own policy priorities. Recent governmental approaches, particularly under Conservative administrations, foregrounded objectives such as stimulating local economies and addressing regional inequalities. This orientation is evident both in the expansion of the devolution deals system and in the quiet abandonment of EVEL—a measure that had sought to rebalance parliamentary representation on explicitly constitutional grounds but ultimately foundered on political and procedural considerations.

Brexit fundamentally altered the constitutional landscape in which these choices were made. It transformed the English Question from a matter of discretionary administrative design into an issue of constitutional necessity. The structural weaknesses embedded within the UK’s territorial constitution—long obscured by the operation of EU law—were exposed with particular force by withdrawal.^40^ In this changed environment, the failure to accommodate England’s distinctive position became not merely anomalous, but destabilising.

The referendum of June 2016 marked not only a political rupture, but a constitutional reckoning. While formally a decision of the UK as a whole, the territorial dynamics of the vote were sharply asymmetrical. England and Wales voted to leave the EU, while Scotland and Northern Ireland voted to remain.^41^ Given England’s demographic scale, it was English preferences that ultimately determined the outcome. Yet, England possessed no institutional forum through which that preference could be articulated or owned as English. Brexit was negotiated, implemented, and justified through the machinery of the UK government, which simultaneously acted as the executive of the Union and, by default, of England.^42^

At first sight, this juxtaposition gives rise to an apparent tension. England was decisive in determining the constitutional direction of the state, yet remained constitutionally unrecognised as a political community in its own right. That tension, however, is not accidental. It reflects precisely the difficulty identified by the Kilbrandon Commission: the problem of governing a Union in which one component is so demographically and politically dominant that its preferences become indistinguishable from those of the state itself. England’s capacity to determine the outcome of the Brexit referendum does not contradict Kilbrandon’s diagnosis, but confirms it. Recognition, on this view, is dangerous precisely because it risks converting latent dominance into explicit constitutional authority.^43^

The argument advanced here does not deny that risk. Rather, it contends that Brexit has altered the conditions under which that risk must be assessed. England’s continued non-recognition no longer functions as a constraint upon its dominance. Instead, it obscures responsibility for its exercise. England shaped the post-Brexit settlement not through institutions accountable to an English electorate, but through UK-wide institutions whose authority is formally undifferentiated. The consequence is not restraint, but diffusion: political power exercised without a clearly identifiable locus of English accountability. In this respect, England’s constitutional absence no longer stabilises the Union in the way Kilbrandon envisaged; it now contributes to instability by blurring the boundary between English preference and UK authority.

This blurring has practical as well as symbolic consequences. England stands alone among the constituent parts of the UK in lacking a system of national devolution. It is governed directly through the central institutions of the state, which simultaneously determine the constitutional parameters within which the devolved administrations operate. Legislation developed by the UK government may apply only to England, yet is not always articulated or scrutinised as such. English interests are therefore projected as default UK-wide norms, reinforcing England’s dominance while denying it distinct political expression.

Prior to Brexit, this arrangement was undoubtedly fragile, but it was partially stabilised by the shared framework of EU membership.^44^ The supremacy of EU law ensured a degree of external constraint and harmonisation that mitigated some of the tensions arising from asymmetric devolution. Withdrawal removed that external scaffold. In its absence, the inadequacy of domestic mechanisms for managing territorial asymmetry—and, in particular, for accommodating England—became starkly apparent.

UKIMA exemplified the operation of these problems. Framed as a technical measure to ensure economic continuity, it imposed the market-access principles of mutual recognition and non-discrimination across the UK.^45^ In practice, this recentralised control in Westminster and, by extension, entrenched England’s regulatory dominance. Because the Act prevents devolved legislatures from modifying its core provisions while leaving Parliament free to do so, the standards set for England inevitably became the baseline for the whole internal market. What appeared as neutral market unity therefore functioned as an extension of English policy preferences through the authority of the UK state.^46^

The implications for England are paradoxical. The Act allowed English choices to define the Union’s economic framework, yet England lacks any institution through which those choices can be debated and legitimised as its own. England’s dominance is exercised by default rather than by design. As the Kilbrandon Commission observed half a century ago, a federation of four units would be ‘so unbalanced as to be unworkable’ because of England’s overwhelming scale and wealth.^47^ That diagnosis remains germane, and it continues to pose a profound difficulty for any attempt to recognise England as a political community within the Union. The argument here is not that recognition would be stabilising in itself, but that the continued absence of any English locus of accountability has become increasingly destabilising in the post-Brexit context. Recognition, if it occurs, would therefore need to be understood not as a solution to the Kilbrandon problem, but as a response to the new forms it has taken.

Neil MacCormick’s account of the UK as an ‘incorporating union’ reinforces the depth of this dilemma. For MacCormick, the absence of meaningful recognition for constituent political communities was a source of structural instability within multinational polities. English dominance within the Union featured prominently in his critique, and it ultimately led him to question the viability of the Union itself rather than to advocate its internal accommodation.^48^ His work is therefore invoked here not as a template for reform, but as a diagnosis of imbalance. The relevance of MacCormick’s analysis lies in highlighting the costs of governing a polity in which one nation’s political weight is exercised through undifferentiated state institutions, obscuring responsibility and intensifying conflict.

Read in this light, MacCormick’s work underscores the precariousness of England’s current constitutional position. England’s preferences are expressed through the authority of the UK state rather than through institutions accountable to an English electorate. This arrangement neither restrains England’s dominance nor provides a mechanism through which it can be politically owned. Instead, it perpetuates a condition in which English influence is both pervasive and unacknowledged, amplifying tensions within the Union while denying England itself a coherent framework of self-government.

Subsequent attempts to recalibrate the Union’s intergovernmental machinery illustrate the persistence of this gap. The intergovernmental system created in 1999 proved ill-suited to managing the stresses of withdrawal.^49^ Its successor, established by the 2022 Review of Intergovernmental Relations, introduced clearer procedures, an independent secretariat and a more regularised tiered structure, but remained dependent on political convention.^50^ The creation of the Council of the Nations and Regions by the Labour government in 2024—bringing together the prime minister, the devolved first ministers and the mayors of England’s combined authorities—offered a modest but symbolically significant innovation. For the first time, English regional leaders entered the formal intergovernmental arena.^51^ Yet, their participation reflects the continuing asymmetry of England’s representation. The mayors speak for localities, not for England as a whole; they operate under powers delegated by Whitehall and without legislatures of their own. The Council’s proceedings rely on consent rather than right, and its outcomes, though politically resonant, have no binding legal effect.

The pattern is therefore one of partial accommodation without constitutional recognition. Brexit forced the UK to confront the costs of England’s institutional invisibility, but has so far produced only administrative and procedural remedies. The reforms to intergovernmental relations and the accompanying programme of English devolution mark a step towards coherence, yet they remain bounded by the logic of delegation. England’s governance continues to depend on instruments that can be altered or withdrawn at will.

In this post-Brexit environment, the failure to provide England with mechanisms of representation, fiscal autonomy and institutional security has become a source of constitutional strain not only for the Union, but for England itself. The challenge is no longer one of balancing devolution across four territories, but one of ensuring that the largest of them possesses a framework of self-government commensurate with its responsibilities. The following section examines how the DCEB seeks to answer that challenge, and whether it moves England from administrative management towards constitutional accommodation.

## 4. The English Devolution and Community Empowerment Bill: Consolidating England’s Absence?

The DCEB constitutes the most comprehensive legislative effort yet to rationalise England’s subnational governance. Introduced in the aftermath of Brexit and amid renewed debates over regional inequality, fiscal constraint and territorial imbalance, the Bill aspires to impose order on what has long been a patchwork of asymmetric and experimental arrangements. It seeks to universalise the devolution-deal model, standardise powers across CCAs and SAs, and establish a coherent statutory framework for the exercise of local leadership throughout England.

The ambition of this measure is undeniable. It signals a political recognition that England’s constitutional invisibility is no longer sustainable, and that the stability of the Union depends in part on a clearer articulation of England’s internal governance. Yet, as this section argues, the DCEB risks consolidating rather than resolving the English Question. By codifying administrative decentralisation without conferring constitutional status, it transforms England’s territorial governance into a system of regulated management rather than genuine self-government.

The Bill’s provisions are therefore examined here through four interrelated constitutional tests: identity, entrenchment, fiscal autonomy and voice. These tests provide a lens through which to assess whether the DCEB moves England beyond the logic of delegation towards a form of devolution capable of generating democratic legitimacy, institutional durability and meaningful participation within the territorial constitution of the UK.

### A. Identity: Constructing a Demos from Above

At the heart of the English Question lies a problem not merely of institutions, but of identity. The DCEB attempts to fill the constitutional void left by England’s lack of self-governing structures by legislating into existence new forms of territorial organisation—CCAs and SAs—created through ministerial order and administrative discretion.^52^ These bodies are intended to rationalise England’s fragmented subnational landscape, to co-ordinate the patchwork of local authorities and combined entities, and to extend the mayoral model pioneered in the city regions. Yet, the Bill’s underlying approach to legitimacy rests upon an enduring constitutional instinct: to construct governance from above and assume that identity will follow.

The design of these new authorities remains fundamentally top-down. Clauses 3–8 empower the Secretary of State to designate areas as CCAs or SAs, to determine their geographical extent and internal constitution, and to authorise transfers of functions ‘where it appears expedient to do so’.^53^ The process privileges administrative coherence over democratic consent. As with the abortive regional assemblies of 2004, identity is treated as a function of institutional architecture rather than its precondition. The House of Lords Constitution Committee has repeatedly cautioned that England’s constitutional arrangements have been shaped by ‘piecemeal and uncoordinated reforms which fail to command democratic legitimacy’,^54^ yet the DCEB contains no mechanism of participatory endorsement—no referendum requirement, no duty of local ratification. Whereas the devolved settlements in Scotland, Wales and Northern Ireland were rooted in popular consent, England’s internal constitution continues to be imposed through statutory fiat.

This absence of a participatory foundation matters because identity cannot be legislated into being. Political communities are not administrative constructs; they emerge from sustained engagement, habit and belonging.^55^ England’s local and regional identities are not blank spaces awaiting definition by ministerial order, but plural and overlapping attachments that must be acknowledged and cultivated.^56^

Empirical evidence underscores this difficulty. Surveys conducted by the British Social Attitudes series since 2014 demonstrate that while a growing number of respondents in England identify primarily as ‘English’ rather than ‘British’, that sense of Englishness remains weakly politicised and unevenly distributed.^57^ As John Curtice and Rachel Ormston observe, English identity has tended to manifest as an expression of grievance rather than of governance—mobilised against perceived external influence, whether from Brussels, Edinburgh or Westminster, rather than channelled through institutions representing England itself.^58^ The DCEB does not engage with this complexity; it presumes that administrative rationalisation will create civic loyalty where little presently exists.

The Bill’s technocratic orientation is particularly evident in its silence on the symbolic dimension of governance. Its language is one of co-ordination and efficiency, not representation or belonging. Vernon Bogdanor has long argued that successful devolution requires not only structures of authority, but also narratives of attachment—‘stories of belonging’ that give constitutional form emotional depth.^59^ The DCEB’s discourse of empowerment and productivity, however modern, offers little that could inspire identification with the new authorities it creates. Their legitimacy will depend on practical performance rather than shared meaning, which may suffice administratively but not constitutionally.

Comparative experience suggests that such institutionalism without identity is fragile. As Arend Lijphart observed, stable multilevel systems rest on pre-existing communities of consent rather than on structural imposition.^60^ In England, the refusal to recognise territorial pluralism has left identity formation to administrative improvisation. Localities are treated as objects rather than subjects of constitutional design—consulted but not empowered. This approach runs counter to the logic articulated by Neil MacCormick’s theory of the incorporating union, which holds that the durability of a multinational state depends on mutual recognition among its constituent communities, not the unilateral inclusion of one within another.^61^ England’s subnational bodies, created by regulation and lacking constitutional standing, cannot claim to be recognised partners within that union; they are instead manifestations of administrative convenience.

The DCEB could have mitigated this democratic deficit by embedding minimal participatory safeguards—local ratification referendums, statutory requirements for local constitutional charters or at least a codified duty of co-design between the Secretary of State and affected authorities. In their absence, the Bill risks creating institutions that are procedurally efficient but existentially hollow: entities that manage localities without representing them. The paradox, as Kenny has observed, is that England’s internal diversity is treated as a problem to be managed rather than a resource to be represented.^62^ Unless the DCEB acknowledges that identity formation is a process of co-creation rather than construction, it may reproduce the historical pattern of English self-government as an administrative fiction—a system that governs everywhere yet represents nowhere.

### B. Entrenchment: Administrative Devolution or Constitutional Settlement

If the question of identity concerns the *who* of English self-government, the question of entrenchment concerns the *how long*. The durability of any devolution settlement depends upon whether its institutions possess a measure of constitutional security or whether they remain mere creatures of ministerial discretion. The DCEB offers little comfort on this point. While presented as the first comprehensive framework for English devolution, it does not alter the foundational fact that English subnational governance remains rooted in delegation rather than devolution.

The Bill’s operative provisions empower the Secretary of State to establish, merge, alter or dissolve CCAs and SAs by regulation, subject only to the affirmative resolution procedure of Parliament.^63^ Clauses 10 and 12 permit ministerial direction over the constitution, membership and functions of these bodies, as well as the transfer or withdrawal of powers previously conferred. Such powers are framed in the broadest discretionary language—‘where the Secretary of State considers it appropriate’—which effectively grants Whitehall the ability to redesign the map of English self-government at will.^64^ The result is a scheme that is organisationally sophisticated yet constitutionally precarious.

This fragility stems from the absence of any meaningful principle of entrenchment. It is important not to overstate the contrast between English devolved institutions and the devolved nations in this respect. Numerous constitutional scholars, including McHarg and McCorkindale, have shown that devolution across the UK remains legally contingent.^65^ The Sewel Convention, even when placed on a statutory footing,^66^ operates as a political expectation rather than a legal constraint,^67^ and Parliament’s capacity to legislate unilaterally in devolved fields has been repeatedly demonstrated.^68^ Devolved autonomy, therefore, is not immune from central override.

The distinction lies instead in constitutional posture rather than absolute security. The Scotland Act 1998, the Government of Wales Act 2006 and the Northern Ireland Act 1998 have each acquired the status of constitutional statutes within the meaning of *Thoburn*,^69^ shielding them from implied repeal and signalling their quasi-constitutional character. They establish devolved institutions as national political communities with legislative authority of their own, operating within a settlement that at least aspires to permanence. By contrast, the DCEB’s framework remains an ordinary statute, modifiable through subsequent legislation or even ministerial regulation.^70^ English subnational bodies continue to exercise powers derived wholly from ministerial delegation, without any comparable claim to constitutional recognition. Even where devolved autonomy is politically fragile, it is constitutionally acknowledged; English devolution remains administratively empowered but constitutionally invisible.

Mark Elliott has argued that English reform continues to oscillate between ‘devolution as delegation’ and ‘devolution as constitutionalisation’, with successive governments preferring the former precisely because it preserves ministerial control.^71^ The DCEB perpetuates this tendency: it codifies decentralisation without rebalancing sovereignty. The Secretary of State remains the constitutional principal, the CCAs the agents. Their existence, powers and boundaries depend on continuing ministerial discretion. Even where the Bill uses the language of permanence—speaking of ‘designated areas’ and ‘empowered authorities’—it refrains from recognising any right to self-government. Devolution, in this form, remains a revocable privilege.

In the broader constitutional context, this is a revealing choice. The UK has long been distinguished by its reliance on parliamentary sovereignty rather than entrenched constitutionalism. Yet, as Alison Young observes, even within this tradition, Parliament has shown a growing willingness to create what she terms ‘constitutional islands’: statutory regimes that, by convention or political expectation, resist ordinary alteration.^72^ The DCEB could have joined this category by acknowledging its constitutional character or by conditioning future amendment on regional consent. Its failure to do so suggests that English devolution remains subordinate not only in law, but also in constitutional imagination.

This absence of legal entrenchment is compounded by the Bill’s silence on political entrenchment. No referendum accompanied its introduction, and no formal consultation mechanism exists to require regional approval for its alteration. By contrast, the devolved nations’ settlements are politically entrenched through the Sewel Convention, notwithstanding its legal fragility. No equivalent convention operates within England. The DCEB, therefore, institutionalises what might be termed devolution without dignity: a framework that invites regional responsibility but withholds constitutional status.

The implications of this are far from abstract. In practice, the DCEB could be repealed, amended or restructured by a future government seeking to streamline spending or reassert control. The absence of a protected status undermines not only the security of the institutions themselves, but also the confidence of the actors within them. Constitutional stability requires that subnational authorities know the ground upon which they stand. Where the rules of the game can be changed at any moment, a meaningful partnership becomes impossible.

Minimal forms of entrenchment could have been achieved without constitutional upheaval. The Bill might, for example, have declared that its principal provisions are not subject to implied repeal, following the model of the European Union (Withdrawal) Act 2018 or the Human Rights Act 1998—both recognised as constitutional statutes despite Parliament’s continuing sovereignty. Alternatively, the government could have adopted a convention that major changes to the English devolution framework will require local consent, mirroring the Sewel principle. Either device would have signalled that English devolution is a matter of constitutional principle, not administrative preference.

The failure to pursue such entrenchment leaves England’s internal governance in a constitutional limbo. The DCEB provides the appearance of permanence while preserving the reality of revocability. In doing so, it repeats the central contradiction of English constitutional reform since 1997: an aspiration to devolution that never escapes the gravitational pull of Whitehall. Without legal or political guarantees of endurance, the DCEB’s institutions may prove no more secure than their predecessors—statutory scaffolds awaiting the next ministerial redesign. As Vernon Bogdanor once observed, ‘constitutional change is not merely the enactment of new laws but the reallocation of authority’.^73^ The DCEB enacts the former while evading the latter.

### C. Fiscal Autonomy: The Autonomy to Do Nothing

If the DCEB falters in granting England’s subnational authorities constitutional entrenchment, it falters even more conspicuously in granting them fiscal autonomy. The tension between decentralisation and control has long defined England’s internal governance. Every reform since the late 1990s has promised local empowerment while preserving the Treasury’s primacy over revenue, borrowing and expenditure. The DCEB continues this tradition. It aspires to empowerment but delivers conditionality; it disperses administrative responsibility but centralises financial authority.

Under the Bill’s framework, the fiscal relationship between devolved bodies and central government remains one of dependency rather than autonomy. Clause 37(2) authorises the Secretary of State to ‘make grants’ to CCAs and Sas, and to attach ‘such conditions as the Secretary of State considers appropriate’.^74^ Nowhere does the Bill provide for statutory ‘funding settlements’; instead, financial support will continue to flow through ministerial grant-making powers, subject to Treasury control. CCAs and SAs possess no general power of taxation, no entitlement to a defined share of national revenues and, under Clause 12, may not borrow on capital markets without the Secretary of State’s consent (which itself requires Treasury approval).^75^ The language is conspicuously permissive, and the constitutional implication unmistakable: fiscal dependency is preserved by design, with ‘empowerment’ operating entirely at the level of delegated discretion.

This model reprises the central flaw of the earlier devolution deals: the autonomy to do nothing. Local leaders are expected to innovate, but without the fiscal means to do so. The resulting gap between authority and capacity risks eroding both accountability and ambition. When the levers of policy are separated from the resources required to operate them, democratic responsibility becomes opaque: voters cannot hold local officials to account for spending or taxation decisions that are, in practice, made elsewhere.

This problem does not stem from any absence of democratic pedigree within English local government. On the contrary, local authorities in England possess deep historical roots and clear electoral legitimacy. The constraint lies instead in the narrowness of political choice permitted by a constitutional settlement in which revenue-raising and borrowing powers are tightly circumscribed. The courts have long recognised this structure. In *R v Secretary of State for the Environment, ex parte Nottinghamshire County Council*,^76^ the House of Lords affirmed Parliament’s authority to impose limits on local taxation, emphasising the breadth of central control over local government finance. In *Hazell v Hammersmith and Fulham LBC*,^77^ the House of Lords reinforced the same logic from a different direction, underscoring the judiciary’s willingness to police local innovation where financial activity exceeds statutory authorisation. Together, these cases illustrate a constitutional order in which democratically elected local institutions exist, but within a framework that sharply constrains their capacity to make meaningful fiscal choices.

PACAC, in its 2022 report on English devolution, was explicit on this point: ‘local authorities in England remain heavily dependent on central government grants, with limited control over either the quantum or the conditions of funding’.^78^ It concluded that ‘the Treasury’s dominance of fiscal policy is the principal constraint on meaningful devolution’.^79^

Comparative evidence underscores how extreme this dependency has become. As PACAC noted, English subnational governments control barely 6% of total public revenue—a lower proportion than any OECD country other than Greece.^80^ In contrast, Scotland and Wales, through their devolved parliaments, now command varying powers of income tax and borrowing, and can rely on relatively predictable block grants under the Barnett Formula. English regions, by contrast, must bid competitively for discretionary ‘Levelling Up’ funds, ‘City Region’ deals or ‘Innovation Zones’, all of which remain subject to Whitehall approval. Fiscal autonomy, in this setting, is less a right than a reward.

Mark Sandford’s research for the House of Commons Library confirms that such competition entrenches inequality rather than reducing it: wealthier or better-organised regions attract more investment, while areas of deprivation remain dependent on intermittent grants.^81^

The constitutional consequences of fiscal dependency are profound. Devolution, as Stephen Tierney reminds us, is not simply the distribution of powers, but the creation of a sustainable balance between self-rule and shared rule.^82^ Where the power to tax and spend remains monopolised by the centre, the appearance of devolution conceals the persistence of central sovereignty. The DCEB’s fiscal provisions illustrate this paradox with particular clarity. By withholding own-source revenues, the Bill ensures that localities are politically responsible for policy outcomes but financially answerable to the Treasury. Such an arrangement substitutes delegated expenditure for autonomous decision making—a logic of control cloaked in the language of empowerment.

A more robust model would require either a guaranteed share of national revenues, distributed through a transparent and formula-based mechanism, or the conferral of limited tax-raising powers on devolved authorities. Proposals for a devolved English income-tax surcharge, a local tourist tax or regional retention of business-rate growth have each surfaced in parliamentary debate, but the DCEB adopts none of them.^83^ Nor does it create any equivalent of the Scottish or Welsh fiscal frameworks, which provide predictability in multi-year settlements and delineate borrowing limits agreed jointly between governments.^84^ Without such instruments, the DCEB risks leaving English authorities in a permanent state of fiscal adolescence—responsible enough to be blamed, but not autonomous enough to act.

Vernon Bogdanor has long argued that fiscal control is the ultimate form of political control.^85^ By that measure, the DCEB’s devolution of expenditure responsibilities without matching revenue autonomy perpetuates England’s constitutional subordination. The Bill may diversify the administration of public money, but it does not diversify the ownership of fiscal power. As long as Whitehall retains the keys to the Treasury, English devolution will remain what it has always been: the art of centralisation by other means.

### D. Voice: England in the Union

The problem of voice—the ability to participate in shared rule on a basis of institutional equality—remains the most elusive dimension of English constitutional development. The DCEB is designed to complete England’s internal framework of self-government, but it offers no mechanism by which that framework can speak for England collectively within the Union. In this respect, England remains constitutionally incomplete: it governs locally, but not nationally. The UK government continues to serve as both the executive for the Union and, by default, the executive for England. This duality blurs the distinction between national and Union competences and perpetuates what the House of Lords Constitution Committee has called ‘England’s absence from intergovernmental relations’.^86^

The election of the Labour government in 2024 brought tentative movement on this front. Seeking to ‘reset’ territorial relations, the new administration established the Council of the Nations and Regions, a standing forum bringing together the prime minister, the devolved first ministers and the mayors of England’s combined authorities. Its creation was presented as a step towards a more balanced and collaborative model of governance—a symbolic break from the Westminster-centric habits of the past. The Council’s inclusion of English regional leaders for the first time does provide a more differentiated voice for England, reflecting its internal diversity and the growing significance of metro-mayoral leadership. In principle, this arrangement complements the DCEB’s structural agenda: the Bill seeks to universalise combined authorities across England and standardise their powers, thereby giving the mayors who sit on the Council a more consistent mandate and territorial reach.

A note of caution is required as to the appropriate constitutional comparator. While the participation of English mayors in intergovernmental forums may invite comparison with the devolved governments, such an analogy would be misleading. Combined authorities and their mayors do not constitute devolved governments in the constitutional sense, nor do they exercise authority grounded in legislative autonomy or popular sovereignty. A closer parallel lies in earlier forms of territorially differentiated administration within the UK, such as the metropolitan counties or Strathclyde Region: bodies with substantial executive responsibilities, but operating within a hierarchical framework of delegation rather than shared rule. The significance of the mayoral presence within the Council of the Nations and Regions therefore lies not in constitutional equivalence, but in what it reveals about the limits of England’s current settlement—extensive administrative responsibility without legislative underpinning, fiscal independence or institutional recognition.

Yet, for all its symbolic value, the Council remains constitutionally fragile. Its proceedings rest on political agreement rather than legal entitlement, its secretariat is located within the Cabinet Office and its conclusions carry only moral or political weight. While Scotland, Wales and Northern Ireland are represented in the Council by national executives that derive authority from their legislatures, the English mayors represent only regions of England, operating under powers ultimately delegated by Whitehall. The Council thus exposes the paradox of England’s position: it has representation without recognition. The mayors who attend its meetings embody fragments of English governance, not England as a constitutional whole. The arrangement amounts to a decentralised substitute for what should be a national voice.

This asymmetry is not a technical inconvenience, but a constitutional fault line. As Stephen Tierney has argued, devolution entails both self-rule and shared rule; the latter is impossible without mechanisms that ensure each constituent unit of the state can participate in decisions affecting the Union as a whole.^87^ England exercises a limited form of self-rule through local governance, but it has no institutional means of engaging in shared rule distinct from the UK government itself. Neil MacCormick’s notion of the incorporating union presupposes reciprocal recognition among self-governing partners; England’s current position subverts this ideal. Its regional voices are acknowledged, yet its collective existence remains unarticulated.

The DCEB and the Council of the Nations and Regions thus point in opposite directions. The former codifies administrative decentralisation within England; the latter introduces England’s sub-regions into intergovernmental dialogue. Together, they begin to sketch the outline of a pluralised England, but they stop short of constitutional integration. Neither provides an English forum of co-ordination or a mechanism for aggregating the interests of the regions into a coherent national position. The UK government continues to dominate the intergovernmental sphere, acting simultaneously as participant and arbiter—a conflation that distorts both process and perception.

Recent commentary by the House of Lords Constitution Committee has urged a principle of ‘positive engagement’ among governments, premised on mutual respect and substantive consultation.^88^ While this shift towards co-operative norms is welcome, it remains dependent on political goodwill rather than legal design.

The persistence of this asymmetry underscores the continuing incompleteness of England’s place within the Union. The DCEB expands the machinery of local governance and the Council gives that machinery a seat at the intergovernmental table, but neither resolves the structural confusion whereby the UK government is both England’s representative and the Union’s government. Without a distinct channel through which English interests can be expressed and reconciled with those of the devolved nations, England remains constitutionally diffuse—present everywhere but represented nowhere. Unless these new arrangements evolve into a more durable system of recognition—one that allows England to act as a participant rather than a proxy—the English Question will persist, not as an omission, but as a structural feature of the UK’s unsettled constitution.

### E. A Qualified Step Forward

The DCEB, together with the Labour government’s wider reforms to intergovernmental relations, represents the most ambitious attempt in a generation to provide England with an internal architecture of self-government. Its ambition, coherence and symbolic reach are not in doubt. For the first time, England’s territorial governance is conceived as a system rather than a sequence of *ad hoc* initiatives. In this sense, the reforms acknowledge—implicitly, if not expressly—that England’s constitutional invisibility has become increasingly difficult to sustain. Yet, measured against the four tests identified above—identity, entrenchment, fiscal autonomy and voice—the reforms remain an exercise in codifying delegation rather than constituting devolution.

In terms of identity, the DCEB advances the project of recognisable local governance, but does so from the centre outward. Its design relies on administrative rationality rather than participatory consent. CCAs and SAs are created through ministerial order, not local choice. This does not deny that institutions can, over time, contribute to the formation of political identity, as the Welsh experience illustrates. But in England’s case, the absence of a prior or parallel process of consent leaves identity as an object of management rather than a source of constitutional legitimacy. The result is an England mapped by Whitehall rather than imagined by its citizens.

On the question of entrenchment, the DCEB’s constitutional modesty remains its defining feature. Its institutions are creatures of statute, revocable by the same authority that created them. No clause insulates their existence from future repeal and no convention guarantees their continued autonomy. This fragility should not be overstated: devolution across the UK remains legally contingent, and even the devolved settlements are vulnerable to unilateral alteration. The distinction lies instead in constitutional posture. While Scotland, Wales and Northern Ireland have evolved into constitutionally recognised entities within the UK’s territorial order, England’s subnational structures remain anchored in administrative discretion. This absence of entrenchment is not merely legal; it is symbolic. It signals that English devolution continues to depend upon political preference rather than constitutional principle.

The problem of fiscal autonomy compounds this fragility. The Bill’s financial provisions replicate the logic of dependence that has long defined English localism. Funding flows from the Treasury through conditional grants, negotiated settlements and time-limited programmes. This constraint does not reflect a lack of democratic legitimacy within English local government, but the narrowness of political choice permitted by a settlement in which revenue-raising and borrowing powers remain tightly controlled by the centre. Without independent sources of revenue or statutory borrowing powers, local authorities exercise responsibility without capacity. The rhetoric of empowerment conceals a continuing hierarchy of control.

The fourth test, voice, reveals both the Bill’s most promising innovation and its most enduring limitation. The creation of the Council of the Nations and Regions, bringing English mayors into regular dialogue with the devolved first ministers, has begun to erode England’s constitutional invisibility. It introduces, for the first time, an institutional mechanism through which English regional leaders can participate in the politics of shared rule. Yet, this development must be understood with care. The participation of mayors does not amount to the recognition of England as a self-governing unit, but reflects a model of representation closer to earlier forms of delegated regional administration than to devolved government. The mayors represent regions, not England as a whole, and their participation rests on political discretion rather than legal entitlement. The Council’s existence is thus an important but incomplete step: it recognises England’s internal diversity without articulating its collective presence.

Taken together, these reforms mark progress in administrative rationality but not yet in constitutional maturity. They make England more *visible* within the Union, but not more *sovereign* within its sphere of competence. The DCEB and the Council together illustrate the incrementalism that defines the UK’s constitutional evolution—change by accretion, not by design. This incrementalism carries benefits: it enables adaptation and avoids rupture. But it also preserves the very ambiguity that, in the post-Brexit context, has become increasingly difficult to justify. England’s governance is simultaneously localised and centralised, plural and unitary, represented and silent.

In constitutional terms, the DCEB may therefore be understood as a *qualified step forward*: a moment of consolidation rather than transformation. It codifies the gains of two decades of English localism, and by institutionalising mayors within national dialogue it begins to correct the democratic deficit identified by successive commissions. Yet, as Kilbrandon cautioned, institutional articulation carries risks as well as possibilities, and nothing in the current reforms resolves the deeper tension between England’s dominance and its absence. England remains governed by the state that it largely constitutes.

If progress is to move beyond managerial refinement, the next phase of reform will require a more deliberate act of constitutional imagination. England’s self-government must be grounded in institutions that derive legitimacy from consent, not convenience; secured by constitutional status, not ministerial discretion; sustained by fiscal independence, not dependency; and represented through mechanisms of shared rule that distinguish the nation from the state. Until those conditions are met, the DCEB will stand as evidence not of constitutional settlement, but of constitutional hesitation—a careful advance that stops short of the recognition required to resolve the English Question.

## 5. Conclusion

This article has traced the evolution of England’s constitutional absence from the hesitant reforms of the late 20th century to the post-Brexit attempts at institutional accommodation. Brexit exposed what decades of administrative improvisation had obscured: that England’s lack of formal recognition within the UK’s territorial constitution is not a technical irregularity, but a structural defect. For much of the devolution era, this ambiguity functioned as a form of containment, limiting the visibility of England’s dominance within an asymmetrical Union. The withdrawal from the EU removed the external disciplines that once masked internal asymmetry, forcing the state to confront directly the question of where England resides within the Union.

The DCEB represents the most concerted attempt yet to answer that question. It offers a rationalised and comprehensive framework for subnational governance, and its combination of territorial standardisation and symbolic inclusion—particularly through the creation of CCAs, SAs and the Council of the Nations and Regions—suggests a genuine effort to bring coherence to England’s fragmented map. Yet, evaluated through the four constitutional tests of identity, entrenchment, fiscal autonomy and voice, the Bill reveals the limits of this administrative turn. It deepens local capacity without displacing central control; it creates new institutions without embedding new guarantees. England’s local bodies remain delegated, dependent and revocable creatures of statute rather than partners in the constitutional order.

The resulting settlement consolidates England’s visibility without resolving its absence. It produces a more organised form of subordination, one that manages England’s diversity but does not represent it. In giving institutional shape to England’s administrative pluralism, the DCEB provides coherence but not autonomy, inclusion but not recognition. The logic of parliamentary sovereignty and the Treasury’s enduring fiscal dominance ensure that England’s governance remains ultimately derivative of the UK’s central institutions.

The persistence of this asymmetry carries consequences beyond England itself. So long as England’s interests are articulated through the UK government rather than through English institutions, the distinction between England and the Union remains blurred. This blurring no longer operates as a neutral stabiliser. Instead, it diffuses responsibility for the exercise of England’s political weight, weakening accountability within England while intensifying mistrust among the devolved nations, whose negotiations are conducted with a partner that is also their competitor. The paradox is stark: England’s constitutional silence sustains the Union’s form while eroding its substance.

The reforms examined here are therefore both significant and insufficient. They mark a threshold rather than a resolution—a recognition that England’s invisibility is untenable, but also a reluctance to transform recognition into autonomy. As Kilbrandon warned, the constitutional articulation of England carries risks as well as possibilities, and nothing in this analysis suggests that recognition would be inherently stabilising. Brexit has not dissolved that dilemma; it has altered the conditions under which it must be confronted. The future stability of the Union may depend less on grand acts of constitutional design than on this more incremental task: the gradual conversion of delegation into self-government, and of administrative presence into constitutional voice. Until that transformation occurs, England will remain what Brexit revealed it to be—not merely dominant, but dominant without ownership, indispensable to the Union, yet constitutionally undefined.

## Footnotes

1 John Mawson, ‘Devolution and the English Regions’ in Gill Bentley and John Gibney (eds), *Regional Development Agencies and Business Change* (Routledge 2000) 26.

2 Public Administration and Constitutional Affairs Committee, *Devolution and Exiting the EU: Reconciling Differences and Building Strong Relationships* (2017–19, HC 1485) <https://publications.parliament.uk/pa/cm201719/cmselect/cmpubadm/1485/1485.pdf> accessed 9 February 2026.

3 House of Lords Select Committee on the Constitution, *The Union and Devolution* (2015–16, HL 149) para 353 <https://publications.parliament.uk/pa/ld201516/ldselect/ldconst/149/149.pdf> accessed 9 February 2026.

4 Public Administration and Constitutional Affairs Committee, *Governing England* (2022–23, HC 463) <https://publications.parliament.uk/pa/cm5803/cmselect/cmpubadm/463/report.html> accessed 9 February 2026.

5 Royal Commission on the Constitution, *Report of the Royal Commission on the Constitution 1969–1973* (Cmnd 5460, 1973) para 531.

6 Neil MacCormick, *Questioning Sovereignty: Law, State, and Nation in the European Commonwealth* (OUP 1999).

7 Electoral Commission, ‘EU Referendum Results’ (*Electoralcommission.org.uk* 2018) <www.electoralcommission.org.uk/find-information-by-subject/elections-and-referendums/past-elections-and-referendums/eu-referendum/electorate-and-count-information> 9 February 2026.

8 See Vernon Bogdanor, *Devolution in the United Kingdom* (2nd edn, OUP 2001); Robert Hazell (ed), *The English Question* (Manchester UP 2006).

9 Michael Kenny, Iain McLean and Akash Paun (eds), *Governing England: English Identity and Institutions in a Changing United Kingdom* (British Academy 2018).

10 See Stephen Tierney, ‘Federalism in a Unitary State: A Paradox Too Far?’ (2009) 19 Regional & Federal Studies 237. See also S Tierney, ‘Brexit and the English Question’ in Federico Fabbrini (ed), *The Law & Politics of Brexit* (OUP 2017).

11
*The Union and Devolution* (n 3) para 288.

12 HC Deb 14 November 1977, vol 939, col 123.

13 Mark Elliott, ‘The McKay Commission and the “West Lothian Question”’ (Public Law for Everyone, 25 March 2013) <https://publiclawforeveryone.com/2013/03/25/the-mckay-Commission-and-the-west-lothian-question/> accessed 9 February 2026.

14 Both the Scotland Act 1978, s 85(1) and the Wales Act 1978, s 80(1) required not only a simple majority vote in favour of devolution, but also that such a majority must constitute at least 40% of the total electorate, thereby setting a high bar for implementation. This threshold was not met in either referendum, leading to the suspension of the legislation.

15
*Governing England* (n 4).

16 ‘In Full: David Cameron Statement on the UK’s Future’ (*BBC News*, 19 September 2014) <www.bbc.co.uk/news/uk-politics-29271765> accessed 9 February 2026.

17 See Robert Hazell and Roger Masterman, ‘Devolution and Westminster’ in A Trench (ed), *The State of the Nations 2001: The Second Year of Devolution in the United Kingdom* (Imprint Academic 2001).

18 Labour Party, ‘1997 Labour Party Manifesto’ (Labour-party.org.uk, 1997) <www.labour-party.org.uk/manifestos/1997/1997-labour-manifesto.shtml> accessed 9 February 2026.

19 Department for Transport, Local Government and the Regions, *Your Region, Your Choice: Revitalising the English Regions* (White Paper, Cm 5511, 2002) <https://dera.ioe.ac.uk/10161/1/Your_region_your_choice_-_revitalising_the_English_regions.pdf> accessed 9 February 2026.

20 RDAs were abolished in 2010 by the Coalition government, in favour of Local Enterprise Partnerships. This signalled a return to centralised and uneven patterns of governance. This policy reversal underscores the vulnerability of English regionalism when not constitutionally protected. For further information on RDAs, see the Regional Development Agencies Act 1998. See also Guy Lodge and James Mitchell, ‘Whitehall and the Government of England’ in Hazell, *The English Question* (n 8) 96–118; Bentley and Gibney (n 1).

21 Office of the Deputy Prime Minister, *Draft Regional Assemblies Bill: Including Explanatory Notes and Regulatory Impact Assessment* (Cm 6285, July 2004) paras 31–35; House of Commons, Office of the Deputy Prime Minister Committee, *The Draft Regional Assemblies Bill* (2004–05, HC 62).

22 See eg *The Union and Devolution* (n 3) chs 2–3.

23 ‘North East Votes ‘No’ to Assembly’ (*BBC News*, 5 November 2004) <http://news.bbc.co.uk/1/hi/uk_politics/3984387.stm> accessed 9 February 2026.

24 For comparative discussion of English options, see Meg Russell and Jack Sheldon, *Options for an English Parliament* (Constitution Unit, March 2018) Report 179.

25 See Tierney, ‘Brexit and the English Question’ (n 10). See also Michael Keating, ‘Brexit and the Nations’ (2018) 90 Political Quarterly 167.

26 See Michael Kenny and Tom Kelsey, ‘Devolution or Delegation? What the Revolt of the Metro Mayors over Lockdown Tells Us about English Devolution’ (Centre on Constitutional Change, 2020) < https://blogs.lse.ac.uk/politicsandpolicy/devolution-or-delegation/ > accessed 9 February 2026.

27 See Council of the Nations and Regions, *Report* (PolicyWise, October 2024) <www.policywise.org.uk/sites/www.policywise.org.uk/files/The%20Council%20of%20the%20Nations%20and%20Regions%20Report.pdf> accessed 9 February 2026.

28
*The Union and Devolution* (n 3) para 56.

29
*Governing England* (n 4); see also Arianna Giovannini, ‘The Uneven Governance of Devolution Deals in Yorkshire: Opportunities, Challenges and Local (Di)visions’ in Craig Berry and Arianna Giovannini (eds), *Developing England’s North* (Springer 2018).

30 Stephen Tierney, ‘Brexit and the English Question’ in Federico Fabbrini (ed), *The Law & Politics of Brexit* (OUP 2017).

31 See Giovannini (n 29).

32 Stephen Tierney, *Constitutional Law and National Pluralism* (OUP 2004) chs 3 and 6.

33 Vernon Bogdanor, ‘Devolving and Not Forgetting’ in Richard Johnson and Yuan Yi Zhu (eds), *Sceptical Perspectives on the Changing Constitution of the United Kingdom* (Hart Publishing 2023) ch 14.

34 Commission on the Consequences of Devolution for the House of Commons (McKay Commission), *Report* (March 2013) <https://researchbriefings.files.parliament.uk/documents/SN06821/SN06821.pdf> accessed 9 February 2026.

35 For a through discussion on the EVEL procedures, see Tierney, ‘Brexit and the English Question’ (n 10).

36 House of Commons Library, ‘English Votes for English Laws (EVEL)’ (2 August 2021) Research Briefing CBP 7339, 7–8.

37 ibid 10.

38 HC Deb 13 July 2021, vol 699, col 1245 (Chloe Smith, Minister for the Constitution).

39 See Robert Hazell, ‘The English Question: Can Westminster Be a Proxy for an English Parliament?’ [2001] PL 268. See also Brigid Hadfield, ‘Devolution, Westminster and the English Question’ [2005] PL 286; Daniel Gover and Michael Kenny, ‘Answering the West Lothian Question? A Critical Assessment of “English Votes for English Laws” in the UK Parliament’ (2018) 71 Parliamentary Affairs 760.

40 See generally Michael Keating, *State and Nation in the United Kingdom* (OUP 2021) 145–7 (on EU law as a framework mitigating asymmetrical tensions). See also Andrew Glencross, ‘Managing Differentiated Disintegration: Insights from Comparative Federalism on Post-Brexit EU–UK Relations’ (2020) 23 British Journal of Politics and International Relations 593.

41 ‘Electoral Commission (n 7).

42 Tierney, ‘Brexit and the English Question’ (n 10).

43 See *Report of the Royal Commission on the Constitution 1969–1973* (n 5).

44 European Communities Act 1972, s 2(4); see also *Thoburn v Sunderland City Council* [2002] EWHC 195 (Admin), [2003] QB 151, which affirmed the constitutional significance of EU law’s supremacy.

45 See UKIMA, ss 2–3 on mutual recognition, ss 5–6 on non-discrimination for goods, and ss 17–20 and ss 21–3 for equivalent provisions on services.

46 See Christopher McCorkindale, ‘Legislative Consent, Constitutional Convention, and Constitutional Ill-Health’ (2024) 75(1) NILQ 83; Thomas Horsley and Jo Hunt, ‘Brexit and the UK Internal Market Act 2020: Domestic Constitutional Dislocation and the Reimagination of Internal Trade’ (2024) 75(1) NILQ 19; Thomas Horsley, ‘Constitutional Reform by Legal Transplantation: The United Kingdom Internal Market Act 2020’ (2022) 42 OJLS 1143.

47
*Report of the Royal Commission on the Constitution 1969–1973* (n 5) para 531.

48 MacCormick (n 6).

49 See Richard Rawlings, ‘Brexit and the Territorial Constitution: Devolution, Reregulation and Intergovernmental Relations’ (2020) 36(1) PL 64; HL Constitution Committee, *Respect and Co-operation: Building a Stronger Union for the 21st century* (10th Report of Session 2021–22, HL Paper 142).

50 ‘Review of Intergovernmental Relations’ (HM Government, January 2022) <www.gov.uk/government/publications/the-review-of-intergovernmental-relations> accessed 9 February 2026.

51 Council of the Nations and Regions (n 27).

52 DCEB, cls 1–8 and sch 23.

53 ibid cl 4(1).

54
*The Union and Devolution* (n 3) para 56.

55 See Michael Kenny, *The Politics of English Nationhood* (OUP 2014) 210–12.

56 See Kenny and Kelsey (n 26).

57
*British Social Attitudes Survey 41* (NatCen 2024) 132–4.

58 John Curtice and Rachel Ormston, ‘Is Englishness Becoming a Political Force?’ in Alison Park and others (eds), *British Social Attitudes: The 33rd Report* (NatCen Social Research 2016) 55–78.

59 Vernon Bogdanor, *The New British Constitution* (Hart Publishing 2009) 164.

60 Arend Lijphart, *Patterns of Democracy* (Yale UP 2012) 203–5.

61 MacCormick (n 6) 118–21.

62 Michael Kenny, ‘The Idea of English Regionalism Revisited’ (2021) Political Quarterly 92(4) 627.

63 DCEB, cls 3–4, 8 and sch 23.

64 ibid cl 12(1).

65 See Aileen McHarg, ‘Brexit and Its Implications for Democratic Governance in the United Kingdom’ in Glenn Patmore (ed), *Research Handbook on Law and Democracy* (Edward Elgar, forthcoming); Aileen McHarg, ‘The Miller Case and the Sewel Convention’ in Mark Elliott, Jack Williams and Alison Young (eds), *The UK Constitution after Miller* (Hart Publishing 2018); Christopher McCorkindale, Aileen McHarg and Paul F Scott, ‘The Courts, Devolution, and Constitutional Review’ (2017) 36 UQLJ289; Oran Doyle, Aileen McHarg and Joe Murkens (eds), *The Brexit Challenge for Ireland and the United Kingdom: Constitutions under Pressure* (CUP 2021).

66 Scotland Act 1998, s 28(8) (as inserted by the Scotland Act 2016, s 2); Government of Wales Act 2006, s 107(6) (as inserted by the Wales Act 2017, s 2).

67
*R (Miller) v Secretary of State for Exiting the European Union* [2017] UKSC 5 [151].

68 European Union (Withdrawal) Act 2018; United Kingdom Internal Market Act 2020; Retained EU Law (Revocation and Reform) Act 2023, each enacted notwithstanding devolved competence and without full legislative consent from the devolved legislatures.

69
*Thoburn* (n 44).

70 Scotland Act 1998, s 63A; Government of Wales Act 2006, s 107; Northern Ireland Act 1998, s 5; *Thoburn* (n 44) [62]–[63].

71 Mark Elliott, ‘Devolution, Delegation and the English Constitution’ (2017) PL 414.

72 Alison Young, *Democratic Dialogue and the Constitution* (OUP 2017) 189–92.

73 Bogdanor, *The New British Constitution* (n 59) 167.

74 DCEB, cl 37(2).

75 ibid cl 12.

76 [1986] AC 240.

77 [1991] 1 AC 521.

78
*Governing England* (n 4) para 18.

79 ibid para 22.

80 ibid para 24.

81 Mark Sandford, *Devolution to Local Government in England* (House of Commons Library, 2023) Research Briefing Paper CBP 7029, 11–12.

82 Tierney, *Constitutional Law and National Pluralism* (n 32) ch 8.

83 HC Deb 7 March 2024, vol 747, col 1123 (Clive Betts MP); see also *Levelling Up, Housing and Communities Committee*, *Financial Scrutiny of Local Government Funding* (2023–24, HC 957).

84 See *HM Treasury and Scottish Government*, *The Fiscal Framework Agreement* (February 2016); *HM Treasury and Welsh Government*, *Fiscal Framework Agreement* (December 2016).

85 Bogdanor, *The New British Constitution* (n 59) 173.

86
*The Union and Devolution* (n 3) para 56.

87 Tierney, *Constitutional Law and National Pluralism* (n 32) ch 8.

88 House of Lords Constitution Committee, *The Union and Devolution: Follow-Up Report* (2nd Report of Session 2023–24, HL Paper 39) para 23.

